# What Is the Role of Giant Endosomal Sorting Complexes Required for Transport (ESCRT) Structures in T Cell Activation?

**DOI:** 10.1002/adbi.202500226

**Published:** 2025-11-22

**Authors:** Anthi Psoma, Femmy C. Stempels, Rinse de Boer, Geert van den Bogaart

**Affiliations:** ^1^ Department of Molecular Immunologygroningen Biomolecular Sciences and Biotechnology Institute University of Groningen Groningen The Netherlands

**Keywords:** Dendritic cell, ESCRT, Immunological synapse, T cell

## Abstract

Our lab recently discovered uniquely large (multiple micrometres) ring‐shaped cellular structures composed of endosomal sorting complexes required for transport (ESCRT) proteins. These structures are formed by tissue infiltrating fibroblasts, dendritic cells (DCs) and macrophages but only in specific culturing conditions, notably in dense three‐dimensional collagen matrices or without serum on glass supports. We also found that the structures are devoid of F‐actin and form at membrane damage sites, suggesting a role of these structures in membrane repair. Another possibility is that these structures have a role in the immunological synapses (IS) with T cells, because they surround clusters of tetraspanins and integrins that have known roles at the IS. Therefore, we tested the hypothesis that giant ESCRT structures are present at the IS between DCs and T cells and contribute to its stability or signaling. Although we occasionally observed enrichment of ESCRT proteins at the interface between DCs, we do not observe this at the IS between human monocyte‐derived DCs (moDCs) and allogenic T cells. Thus, our data do not support a role for the giant worm‐shaped ESCRT structures at the IS, and suggest that they solely are involved in plasma membrane stability and integrity.

## Graphical abstract

The article explores the discovery of unusually large, ring‐shaped ESCRT protein structures formed by immune and stromal cells. It investigates their formation, composition, and potential roles in immune synapse formation. The findings challenge assumptions about their function in immunological synapses, suggesting an exclusive role in maintaining plasma membrane integrity.

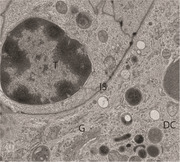

## 1 Introduction

The ESCRT system is composed of four evolutionarily conserved protein complexes, called ESCRT‐0, ‐I, ‐II, and ‐III, that each play distinct functional roles. ESCRT‐0 proteins, including hepatocyte growth factor‐regulated tyrosine kinase substrate (HRS), bind to ubiquitin moieties that are attached to membrane proteins destined for degradation, and thereby are responsible for the first sorting step in the multivesicular body (MVB) pathway. ESCRT‐I proteins, including tumor susceptibility gene 101 (TSG101), and ESCRT‐II proteins form the so‐called core machinery complexes, which together with ALG‐2‐interacting protein X (ALIX), an accessory protein of the ESCRT complex, orchestrate protein sorting and vesicle biogenesis. For instance, small (<0.3 µm^2^) assemblies of ESCRT‐II proteins mediate the formation of intraluminal vesicle (ILVs) in MVBs by deforming and separating the membrane to bud small (<100 nm) vesicles inward into the endosomal lumen [1]. Moreover, ESCRT‐III and the vacuolar protein sorting 4 (VPS4) protein operate together for different membrane remodeling events and are responsible for membrane scission [2]. The ESCRT‐III complex is mainly comprised of the charged multivesicular body proteins (CHMPs) CHMP4A‐C, CHMP2A, CHMP2B, CHMP3, and CHMP6 and the increased sodium tolerance 1 protein (IST1) [3]. In addition to the sorting of ubiquitinated membrane proteins by formation of ILVs in MVBs, multiple other cellular functions of the ESCRT system have been reported over the years, including lipid bilayer remodeling, autophagy, and membrane fission. Moreover, studies have shown that ESCRT proteins play a critical role during membrane repair of lysosomal membranes [4, 5], the plasma membrane [6], and the nuclear membrane [7].

Our lab recently reported that clusters of extremely large (multiple µm‐sized) ring‐ and worm‐shaped ESCRT‐III structures are formed at the plasma membrane of macrophages, dendritic cells (DCs), and fibroblasts when cultured in specific conditions, notably in dense collagen matrices and on glass supports in absence of serum [8]. These structures are composed of multiple ESCRT proteins, including IST1, CHMP1, CHMP4 and TSG101 (Table 1). The structures are devoid of F‐actin and phosphoinositide lipids, but surround clusters of integrins, GPI‐anchored proteins, and tetraspanins. The structures might be a response to a loss of membrane integrity, because the absence of serum (i.e. the condition in which the structures are formed on glass supports) results in very stable integrin‐mediated cell adhesions that potentially cannot dissociate, possibly resulting in membrane damage upon migration. In line with this, the ESCRT structures are located exactly surrounding the integrin‐mediated adhesions. Moreover, the structures also form at the contact sites with membrane‐disruptive silica crystals, in accordance with previous findings that the ESCRT system mediates plasma membrane repair [9]. In addition, the structures were observed at the nuclear membrane when DCs were cultured in dense collagen matrices, a condition which is known to exert forces on the nucleus [10] and thereby potentially also membrane rupture. These findings strengthen the hypothesis that the giant ESCRT structures function in plasma membrane repair and/or as supporting scaffold structures protecting the membrane in cells infiltrating into dense tissues. However, this has not been demonstrated directly in the manuscript and remains a hypothesis.

**TABLE 1:** Proteins located at giant ESCRT structures.

Function	Protein name	Colocalization within the IST1 structures [8]	Localization at the IS in MLR reaction (this study)
ESCRT‐I complex	TSG101	Yes	Occasionally only in 1 donor combination
Regulation of microtubules	Spastin	Yes	No
ESCRT‐III complex	CHMP4	Yes	No
ESCRT‐III complex	IST1	Yes	No
ESCRT accessory protein	ALIX	Yes	No

Alternatively, or additionally, the giant ESCRT structures might also exert completely different functions. One possible alternative function raised by [11] is that the giant ESCRT structures play a role at the immunological synapse (IS) with T cells [11]. The IS is a well‐studied supramolecular protein cluster located at the interface between T cells and antigen presenting cells (APCs) like DCs, macrophages, and cells targeted for cytotoxicity. The IS is full of interacting molecules that are precisely orchestrated in time and place to ensure proper activation of antigen‐specific T cells [12]. There are multiple signals that lead to T cell activation during IS formation. The main signal is related to antigen recognition by the TCR‐MHC‐peptide complex, followed by F‐actin filament assembly. The second signal comes from co‐stimulatory molecules CD80/86 that are present on the surface of the APCs and interact with CD28 on the T cell side. The third signal is controlled by cytokine secretion by APCs on T cell receptors [13]. Finally, interactions of integrin LFA‐1 with Intercellular Adhesion Molecule 1 (ICAM‐1) are responsible for a stabilized synapse. The interactions can lead to a bull's eye structure, with central TCR‐MHC clusters that are surrounded by ICAM‐1—LFA‐1 interactions [14]. Some of the stimulatory molecules share common trafficking routes with MHC molecules to the plasma membrane side [15]. During IS formation, the microtubule organizing center (MTOC) is also translocated to the IS and maintains sustained and controlled T cell signaling [16].

As the giant ESCRT structures were present in dendritic cells and macrophages and not in T cells [8], it seems most likely that if the ESCRT structures are present at the IS, it will be at the side of the APC rather than at the side of the T cell. Based on the known functions of the proteins involved in the giant ESCRT structures, there are multiple possible functions of ESCRT structures at the IS.

Firstly, the ESCRT structures might prevent or repair plasma membrane damage at the IS. In MC38 colorectal cell lines that were cocultured with T cells, it was found that the ESCRT machinery can prevent their killing by cytolytic T lymphocytes (CTLs) [17]. This killing is mediated by perforin that results in the entry of granzyme B and loss of membrane integrity. However, this is prevented in the cancer cells by ESCRT‐mediated repair of the perforin‐induced membrane pores. Possibly, ESCRT proteins protect APCs from T cells too.

Alternatively, the ESCRT structures might form or maintain a strong compact scaffold between the two cells forming an IS. The ESCRT structures are wrapped around clusters of αµ and β2 integrins [8], and integrin signals are well known to contribute to the stabilization of the contact site between the T cell and the APC and thereby facilitate T cell activation, proliferation and cytokine secretion [18]. Together with integrin αL, integrin β2 forms LFA‐1, the major integrin of T cells, which binds to ICAM family proteins. Binding of LFA‐1 to ICAM‐1 is the predominant receptor–ligand interaction responsible for sustained T cell–APC adhesion [19] and these interactions strengthen antigen presentation [20]. This raises the possibility that the giant ESCRT structures somehow are involved in connecting the cells together at the IS by regulating integrin‐mediated cell attachment.

Lastly, ESCRT structures might play a role in the activation and/or differentiation of T cells. ESCRT proteins are well known to mediate the formation of extracellular vesicles [21], raising the possibility that the giant ESCRT structures might release so‐called trans‐synaptic vesicles (tSV), from the APCs to the T cells at the IS. These tSVs might for example contain antigen, immune‐stimulatory or repressive signaling factors and/or differentiation signals. Although the formation of tSVs at the APC side have not been described, several different tSVs are formed at the T cell side of the IS, including polarized exosomes (PE) that are released from MVBs into the synaptic cleft [22], and synaptic ectosomes (SE) that bud from the T cell plasma membrane into the synaptic cleft [23]. These extracellular vesicles are actively formed by the T cells through the action of the ESCRT system [22]. For example, artificial APCs consisting of bead‐supported lipid bilayers presenting surrogate antigen, adhesion, and co‐stimulatory receptors could induce tSV biogenesis. Time‐lapse imaging revealed that PD‐L1 trans‐presentation together with the ESCRT‐I protein TSG101, metalloprotease ADAM10 and tetraspanin CD81 are key regulators in the formation of tSVs carrying the signaling receptor CD40L [23]. Since the giant ESCRT structures also contain TSG101, it might well be that they mediate tSV formation at the APC side of the IS.

An alternative way in which ESCRT structures might affect T cell activation, is via tetraspanins. The giant ESCRT structures surround clusters of the tetraspanin CD63 [8]. Tetraspanins have well known immune signaling functions. For example, immune cells including CD4^+^ T cells, CD8^+^ T cells, and DCs express high levels of tetraspanin CD53 and this is critical for immune cell functions including adhesion, migration, signaling [24]. Tetraspanins are also involved in the IS and function in APCs [25]. For example, it has been proposed that the tetraspanin CD9 mediates lateral association of MHC class II molecules on the DC surface and that tetraspanin CD37 promotes DC migration [26, 27]. Moreover, CD63 was characterised as a crucial activation protein on T cells by an unidentified structure expressed on APCs, because inhibition of CD63 blocks the T cell response to DCs [28]. It might thus well be that the functions of these tetraspanins are somehow related to the giant ESCRT structures. Moreover, tetraspanins are well known cargos of exosomes and are sorted through an ALIX‐ and ESCRT‐III–dependent pathway [29], so they might be sorted into tSVs as well.

Interestingly, in T cells, ESCRT proteins play crucial roles in the control of T cell activation. For example, CHMP5, belonging to the ESCRT‐III family, is a key sensor of thresholds for signaling via the T cell receptor, essential for T cell activation [30]. In addition, TSG101 locates at the IS in T cells, where it plays a role in the formation and the function of the central supramolecular activation cluster (SMAC) [31]. Another interesting finding is that the T cell receptor becomes ubiquitinated in activated T cells and the ubiquitinated receptor is subsequently recognized by HRS. This in turn activates the assembly of clathrin, leading to the routing of the T cell receptors into SEs that can be released from the T cell membrane towards the APCs [32]. It might be that ESCRT proteins exert similar roles at the APC side of the immunological synapse and thereby regulate reverse MHC signaling; a process in which ligation of MHC molecules induces signal‐transduction and cell regulatory effects in the APC [33].

In this study we propose that ESCRT structures might be present at the IS, as they could function to (A) prevent or repair membrane damage, (B) form or maintain a strong compact scaffold between the cells, and/or (C) play a role in the activation and/or differentiation of T cells. We tested whether the giant ESCRT structures are present at the IS between DCs and T cells. However, we did not observe the formation of the structures at T cell‐DC interface formed in the mixed lymphocyte reaction (MLR). Thus, our data do not support a role of the giant ESCRT proteins at the immunological synapse, but instead suggest that they solely play a role in IS‐independent membrane repair and/or scaffolding.

## 2 Results

### 2.1 IST1 Accumulates at the Interface Between DCs

**FIGURE 1:**
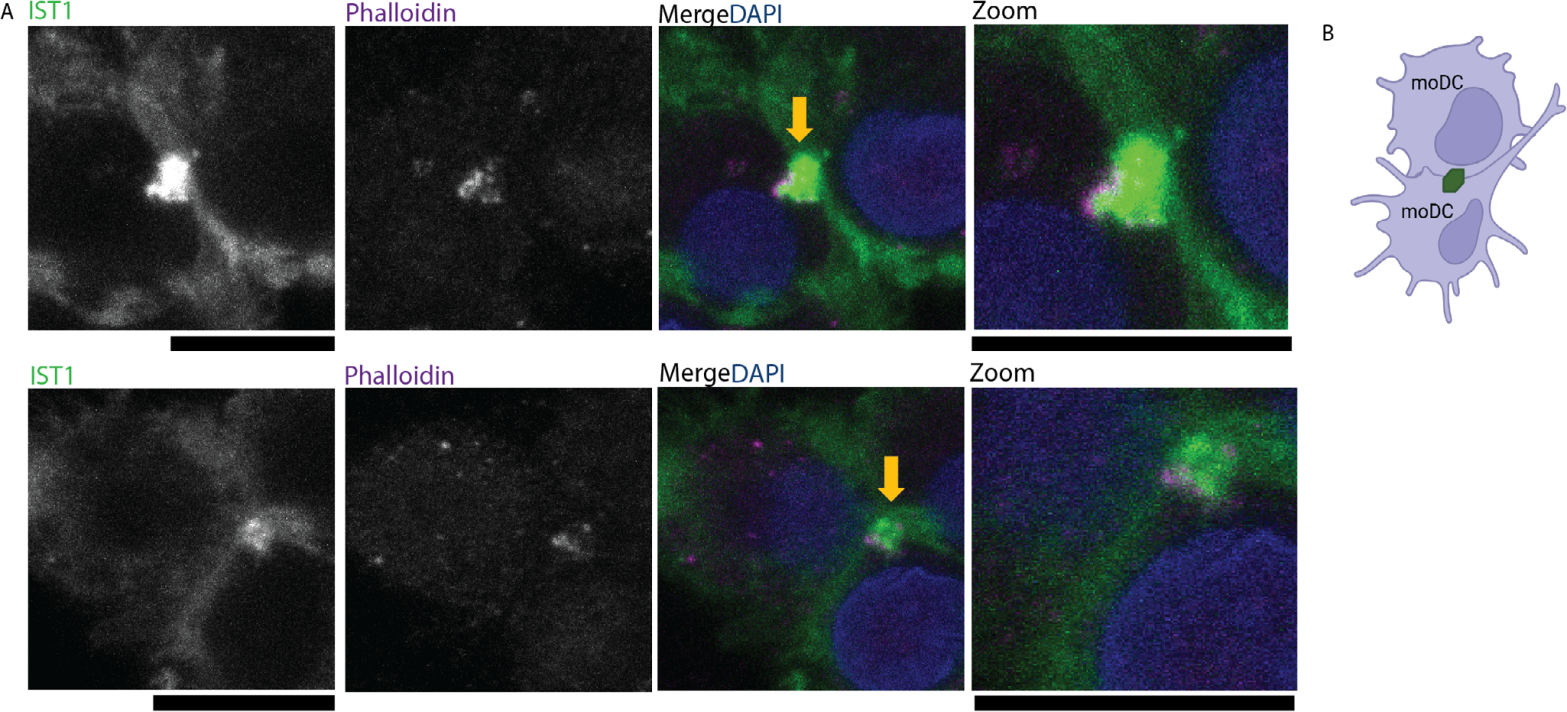
ESCRT protein IST1 accumulates at the interface between dendritic cells. (A) Confocal micrographs of moDCs cultured in dense 3D collagen matrices. Green in merge: IST1, magenta: phalloidin; blue: DAPI. Yellow arrow: ESCRT recruitment between two moDCs. Scale bars: 10 µm. IST1 accumulation at the interface between moDCs was occasionally observed in <5% of DC interfaces. Two examples shown. (B) Schematic graph showing the accumulation of ESCRT protein (green) at the contact site between moDCs.

We previously discovered the presence of micrometer‐sized worm‐ and ring‐shaped ESCRT structures surrounding clusters of integrins in certain tissue‐infiltrating cell types; DCs, macrophages and fibroblasts [8]. Next to binding to ECM, integrins also mediate cell‐cell interactions. Reanalysis of microscopy data that we obtained for our original study [8], revealed the presence of extensive IST1 recruitment occasionally at the interface between two moDCs cultured in collagen matrix (Figure 1A,B). Although we observed this infrequently (<5% of cell‐cell interfaces), it suggests that the giant ESCRT structures could be involved in interaction between the DCs.

### 2.2 Giant ESCRT Structures Are Absent at the IS With Allogenic T Cells

Next, we tested the hypothesis that the giant ESCRT structures are formed at the IS. We cultured moDCs together with peripheral blood lymphocytes (PBLs; ≈80% T cells) from different donors in serum free medium on glass supports. Flow cytometry indicated that the presence or absence of serum did not affect the surface levels of MHC class I and II molecules, nor of the costimulatory receptor CD86 (Figure S1). Co‐culturing moDCs with PBLs results in a so‐called mixed leukocyte reaction (MLR), where moDCs form an IS with the allogenic T cells due to MHC haplotype mismatch. Eventually, the IS formed in an MLR results in the activation of the T cells and, depending on the extent of HLA mismatch, the perforin‐ and granzyme‐mediated killing of the DCs. MLR reactions were performed on glass supports instead of in 3D collagen matrices, as this facilitates the interaction between the adherent moDCs and suspension T cells, which is required for IS formation.

**FIGURE 2:**
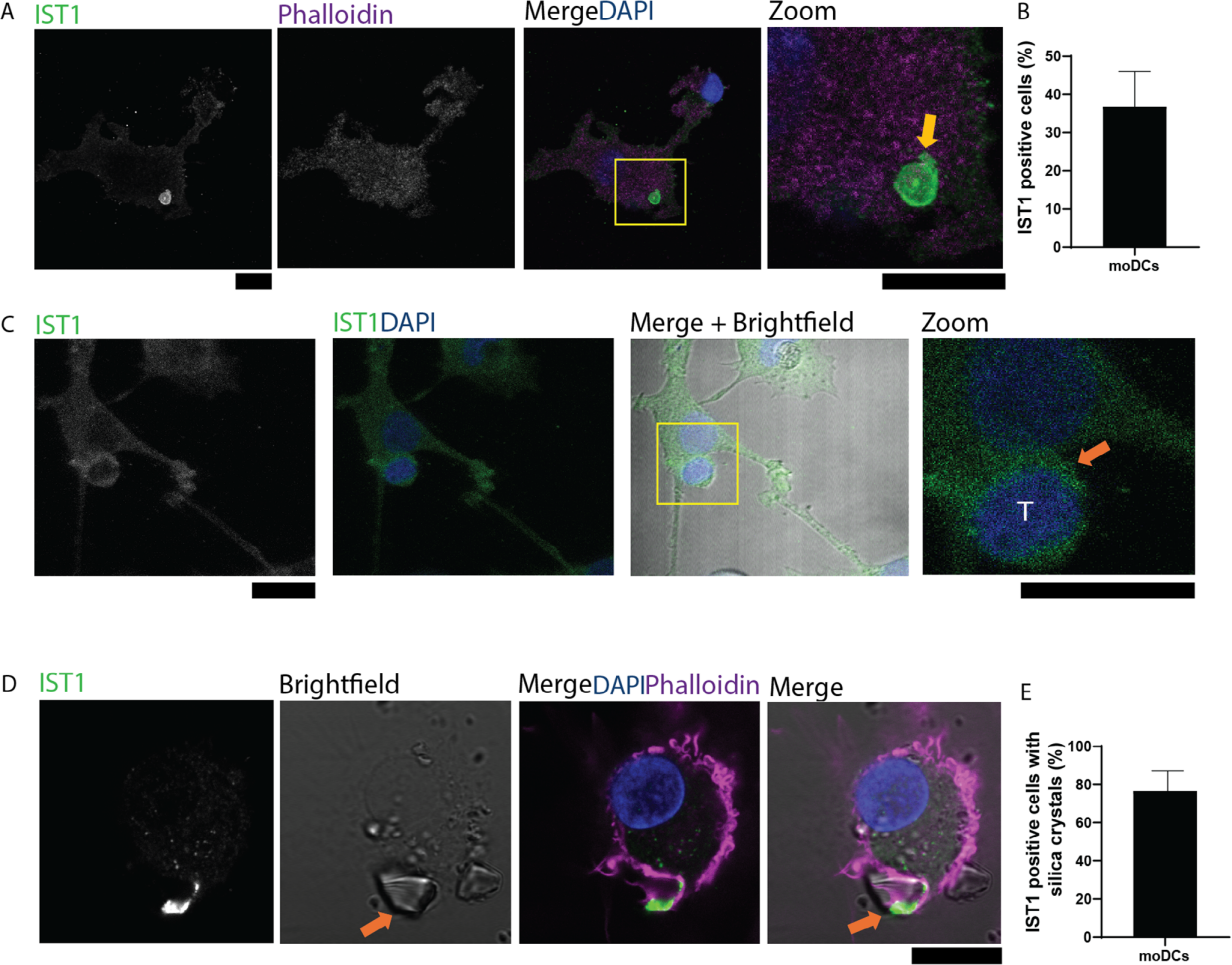
IST1 accumulates at the interfaces of moDCs with membrane disrupting silica crystals but not with allogenic T cells. (A) Representative confocal micrograph of moDCs cultured on glass supports (control) without serum and immunostained for IST1. Green in merge: IST1; magenta: phalloidin; blue: DAPI. Yellow box: zoomed areas. Yellow arrow: giant ESCRT structure. (B) The graph shows the percentage of cells showing IST1 structures (n = 3 independent donors, mean value with SEM of 52 cells). (C) Representative confocal micrographs of moDCs derived from the same donor as in panel A, cocultured with allogenic PBLs on glass supports without FBS. Cells were immunostained for IST1. Green in merge: IST1; magenta: phalloidin; blue: DAPI. Yellow box: zoomed area. Orange arrow: position of the IS (n = 4 independent donors, 23 synapses). (D) Representative confocal micrograph of moDCs cultured on glass support without serum and with membrane‐disrupting silica crystals (orange arrow) after 1 h. Cells were immunostained for IST1. Green in merge: IST1; magenta: phalloidin; blue: DAPI. Scale bars: 10 µm. (E) The graph shows the percentage of cells showing IST1 structures at the interface with silica crystals (mean value with SEM of 20 cells in total).

After 2 h of co‐culturing of the moDCs and the T cells, the cells were fixed and immunostained for IST1. Similar to our previous findings [8], the giant ESCRT structures were apparent at the plasma membrane facing the glass supports, but only in the moDCs (Figure 2A,B).

However, we did not observe accumulation of ESCRT proteins at the interface between moDCs and PBLs, for multiple blood donor combinations (Figure 2C). We also performed time‐lapse live cell fluorescence microscopy with moDCs overexpressing IST1 fused to GFP [8], which were co‐cultured with allogenic PBLs in serum free medium. We used a 63x objective as our goal was to visualize formation of the giant ESCRT structures with the IST1‐GFP plasmid at the synapse. Although we again observed the giant ESCRT structures on the glass support, IST1‐GFP did not accumulate at the IS (Movie S1). Moreover, we observed cell death of the moDC after several hours which could be caused by the allogenic immune response, although we cannot exclude phototoxicity effects.

As control for IST1 staining at cellular interfaces, we performed experiments with 5–10 µm sized membrane disrupting silica crystals. As already shown by Beckwith et al. [9], ESCRT proteins are recruited to the interfaces with those T cell‐sized crystals. We indeed noticed the recruitment of the ESCRT structures to the plasma membrane contact sites with the crystals (Figure 2D,E).

**FIGURE 3:**
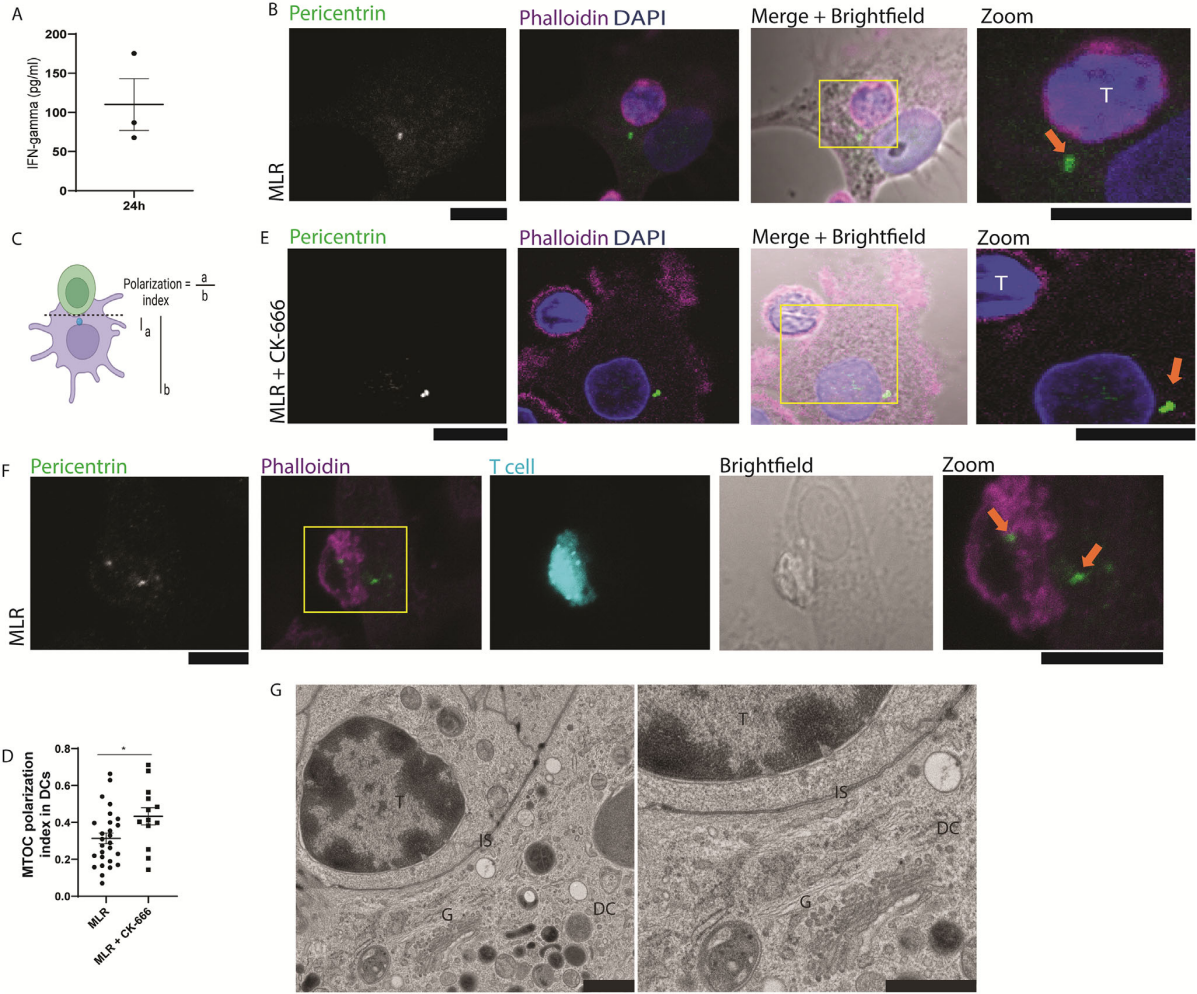
Despite absence of IST1, allogenic T cells form a functional IS with moDCs. (A) Dendritic cells were cultured with allogeneic T cells. After 24 h, culture supernatants were collected for performing ELISA. IFN‐γ production was measured from 3 different donors. (B) Representative confocal micrograph of moDCs cocultured with allogenic PBLs on glass supports. Cells were immunostained for pericentrin. Green in merge: pericentrin; magenta: phalloidin; blue: DAPI. Yellow box: zoomed area. Orange arrow: position of pericentrin close to the IS (n = 3 independent donors, 24 cells). (C) Scheme of quantification of MTOC polarization: distance between the MTOC and the DC‐T cell interface (a) was divided by the diameter of the DC (b). (D) Quantification of MTOC polarization index in dendritic cells in a MLR reaction without/with the ARP2/3 complex inhibitor CK‐666 (*p = 0.0299, two‐tailed unpaired Student's t‐test). (E) Representative confocal micrograph of moDCs cocultured with allogenic PBLs together with CK‐666 on glass supports. Cells were immunostained for pericentrin. Green in merge: pericentrin; magenta: phalloidin; blue: DAPI. Yellow box: zoomed area. Orange arrows: position of pericentrin (n = 3 independent donors, 13 cells). (F) Representative confocal micrograph of moDCs cocultured with PBLs on glass supports. PBLs were stained with Cell Trace Violet (cyan) and cells were immunostained for pericentrin (green). Magenta: phalloidin. (G) Representative transmission electron microscopy of moDCs cocultured with allogenic PBLs. Indicated are the positions of the T cell (T), moDC (DC), IS, and Golgi (G).

Although we cannot differentiate the formation of an IS from mere scanning of the surface of the DC by the T cells in the microscopy experiments, we performed several control experiments to validate IS formation. First, we measured the interferon‐γ (IFN‐γ) in the supernatant of the MLR reactions by ELISA for verifying the formation of a proper synapse (Figure 3A). Second, we assessed the translocation of the MTOC to the IS, by labelling for the MTOC component pericentrin (PCNT), demonstrating formation of productive IS (Figure 3B–F). Third, we labeled the PBLs with CellTrace Violet to confirm synapse formation between the moDCs and the PBLs (Figure 3F). Fourth, we confirmed that MTOC translocation could be blocked by the Arp2/3 inhibitor CK‐666 (Figure 3D,E), in line with the reported role of Arp2/3 in IS formation [34]. Fifth, we performed transmission electron microscopy showing a tight cellular interface between the PBLs and the moDCs as well as localization of the Golgi apparatus in close proximity to this interface (Figure 3G). Finally, we showed using flow cytometry that for some of the allogenic moDC – PBL combinations, the CD3‐negative cells were efficiently killed (Figure S2A,B).

We previously reported that the giant ESCRT structures contain many different ESCRT proteins [8]. We therefore also tested for the presence of other ESCRT proteins at the IS by immunolabeling (Table 1). Next to IST1, the proteins that we assessed were the following: CHMP4A, microtubule severing protein Spastin, ALIX, and TSG101, which are all proteins previously shown to locate in the giant ESCRT structures [8]. These proteins were also not enriched at the IS (Figure S3A–C). However, we occasionally (<5% of all IS) observed the recruitment of TSG101 at the IS, but only in a single allogenic donor combination (Figure S3D). Moreover, we cannot discern whether TSG101 is enriched at the APC side, T cell side, or both with our diffraction limited microscopy.

## 3 Discussion

In this study we explored whether giant ESCRT structures are formed at the IS between DCs and allogenic T cells. We did not observe the accumulation of IST1 and almost all other tested ESCRT proteins at the IS. Despite this being a negative result, we believe our conclusion that the giant ESCRT structures are not recruited to the IS is warranted, because we confirmed productive IS formation by MTOC polarization, IFN‐γ secretion, electron microscopy, and cell killing, and we could detect IST1 accumulation at the cellular interface with membrane disrupting silica crystals. These control experiments demonstrate that we did not overlook IST1 recruitment due to a technical limitation.

However, we sporadically observed an enrichment of the ESCRT protein TSG101 at the IS. It was previously reported by [17] that TSG101 is recruited to the IS in target cancer cells that are attacked by CTLs, following CTL‐mediated perforin and granzyme release [17]. Therefore, TSG101 in cancer cells might counteract the perforin‐induced membrane damage, and thus act as a cellular defense mechanism against CTL induced killing. In DCs, the accumulation of TSG101 might have a similar function and prevent the killing by allogenic CTLs. However, we only observed the enrichment of TSG101 in a small fraction of the synapses and only for a single allogenic donor combination.

Although giant ESCRT structures were not present at the IS, we did observe extensive ESCRT recruitment at the interface between DCs. Given the role of ESCRT proteins in membrane remodeling, it might well be that ESCRT structures are somehow involved in the macrophage fusion for formation of foreign body giant cells (FBGCs). FBGC formation is observed in foreign body responses, and it is proposed that the fusion of macrophages facilitates the phagocytosis of particles that are too large to ingest for single macrophages [35].

A question is why we only observed TSG101 enrichment in a single allogenic donor combination. Another question is why only TSG101 and not other ESCRT proteins seem enriched at the IS, even though other ESCRT proteins are also involved in membrane repair [17]. One possibility is that perforin‐induced pores only cause limited ESCRT‐mediated membrane repair in DCs. Compared to the unregulated membrane rupture caused by silica crystals or laser scission, perforin‐mediated membrane pores are relatively structured with 19 to 24 perforin monomers lining a ≈16‐nm sized pore [36]. Thereby, perforin might prevent or limit the formation of large ESCRT structures, although this remains an untested hypothesis. Another possibility is that the perforin‐induced pores are confined to the IS, and the influx of cations that signal membrane damage, like calcium and potassium, is insufficient to signal membrane damage. This might well be the case, as diffusion can be expected to be limited in the narrow cleft between the DC and T cell membranes. Indeed, we recently showed that close membrane apposition in phagosomes limits the diffusion speed of cations by orders of magnitude [37].

A limitation of this study is that we only looked at the IS between allogenic PBLs and DCs. We selected this type of IS, because both CD4^+^ helper and CD8^+^ killer T cells are engaged in an MLR [38]. However, we cannot exclude the possibility that the giant ESCRT structures function in the formation of specific signaling entities which are not present in this type of IS, but are only present in other types of synapses like with B cells, NK cells, or autologous antigen‐specific DC‐T cell synapses. Similarly, giant ESCRT structures might also be formed in the inhibitory synapses between CTLs with healthy cells and/or CTLs with resistant cancer cells. Another aspect is the duration of the IS, which can last from several minutes (between CTLs and target cells) to several hours till days (between T‐helper cells and APCs) [12]. This duration might also determine the molecules that can be found at the synapse.

All in all, based on our previous study [8] and on the findings in this study, we propose that giant ESCRT structures solely play roles in membrane repair and/or scaffolding, but not in the activation of T cells at the IS.

## 4 Materials and Methods

### 4.1 Cell Culture

Experiments were performed using moDCs and PBLs. Approval to conduct experiments with human blood samples was obtained from the Dutch blood bank, and all experiments were conducted according to national and institutional guidelines. Informed consent was obtained from all blood donors by the Dutch blood bank. Samples were anonymized and none of the investigators could ascertain the identity of the blood donors (NVT0459.00; 15 June 2018). Briefly, peripheral‐blood mononuclear cells (PBMCs) were isolated from buffy coats by density gradient centrifugation. PBLs were separated from monocytes after positive selection of CD14^+^ cells with CD14 microbeads, LS Column and a MidiMACS Separator (Miltenyi Biotec Order no. 130‐050‐201). Monocytes were differentiated into moDCs by culturing in RPMI1640 medium supplemented with L‐glutamine (21875‐034; Gibco), 10% fetal bovine serum (FBS; 10309433; Thermo Fisher Scientific), 1% antibiotic antimycotic (AA; 15240062; Gibco), 300 U/ml interleukin‐4 (130‐093‐924; Miltenyi), and 450 U/ml granulocyte macrophage colony‐stimulating factor (130‐093‐867; Miltenyi) for 6 days at 37°C and 5% CO_2_.

### 4.2 Mixed Lymphocyte Reaction (MLR)

MLRs were performed by coculturing moDCs with PBLs. For the allogenic MLR reactions, human PBLs were obtained from different donors than the moDCs. The MLR assays were carried out on 12 mm diameter glass coverslips in 24‐well plates for 2 h to ensure efficient moDC‐T cell contact in a 1:10 moDC to PBL ratio. Cells were cultured in RPMI1640 serum free medium supplemented with L‐glutamine, 1% antibiotic antimycotic in a total volume of 500 µL per well. For Arp2/3 complex inhibition experiments were performed by adding 20 uM CK666 (CK‐666;442633‐00‐3; Sigma‐Aldrich). moDCs were preincubated for 1 h with CK‐666 prior to co‐incubation with PBLs.

### 4.3 Transfections

MoDCs were transfected by electroporation using the NEON Transfection System (Invitrogen) with 100 µL NeonTips (MPK10025; Thermo Fisher Scientific). The protocol entailed 2 pulses of 1,000 V and 40 ms. The concentration of DNA used was 3 µg per 1 million cells. The IST1‐GFP construct was generated previously [8] and is available at Addgene (#186298).

### 4.4 Immunofluorescence Microscopy of Fixed Cells

50,000 moDCs together with 500,000 PBLs were seeded on 12 mm diameter glass coverslips in 100 µL RPMI 1640 lacking phenol red and FBS and were incubated at 37°C and 5% CO_2_ for 2 h. For Figure 3G, the PBLs where further stained with the dye CellTrace Violet (Invitrogen, Cat. No. C34557), according to the manufacturer's protocol. Subsequently, they were fixed using 4% paraformaldehyde (PFA) for 15 min at room temperature (RT). The coverslips were washed with PBS followed by permeabilization and blocked in PBS with 20 mM glycine, 3% (w/v) BSA fraction V 7.5% (BSA;15260037; Gibco), and 0.1% (w/v) saponin for 1 h at RT. Cells were incubated with primary antibody in this blocking solution overnight at 4°C (Table 2). Subsequently, the cells were washed with PBS three times, and coverslips were incubated for 1 h at RT with a combination of the secondary antibodies donkey anti‐rabbit IgG (H+L) Alexa Fluor 488 (A21206; Thermo Fisher Scientific) and donkey anti‐mouse IgG (H+L) Alexa Fluor 488 (A21202; Thermo Fisher Scientific), donkey anti‐rabbit 647 (A31573; Thermo Fisher Scientific) all at 1:400 dilution. For actin labelling, phalloidin Alexa Fluor 647 (A22287; Thermo Fisher Scientific) and Alexa Fluor 546 (A22283, Thermo Fisher Scientific) were used at 1:200 dilution in parallel with the labeling with secondary antibodies.

**TABLE 2:** Primary antibodies used for samples for confocal imaging and Flow cytometry.

Antibody	Host	Dilution for microscopy	Supplier	Catalog nr
ALIX	Mouse	1:100	BioLegend	634502
CHMP4A	Rabbit	1:100	Thermo Fisher Scientific	PA5‐49410
IST1	Mouse	1:100	Proteintech	66989‐1‐lg
Spastin	Rabbit	1:100	Novus Biological	NBP1‐90230
TSG101	Mouse	1:50	BD	BD61296
Integrin beta 2	Mouse	1:200	Gift from Carl Figdor	
IST1	Rabbit	1:1000	Bethyl	A305‐411A
Pericentrin	Rabbit	1:100	Abcam	ab99341
HLA‐DR, DP, DQ‐APC	Human	1:50	REAfinity™ Miltenyi Biotec	130‐123‐843, 130‐124‐037
HLA‐ABC‐APC	Human	1:50	REAfinity™ Miltenyi Biotec	130‐120‐569, 130‐120‐429
Cd86‐PE	Human	1:50	REAfinity™ Miltenyi Biotec	130‐116‐160, 130‐116‐263
CD3 APC	mouse	1:100	eBioscience	Cat 17‐0038‐42

For the IST1 immunolabeling, the cells were fixed using 4% PFA for 15 min at RT. The coverslips were then washed with PBS followed by permeabilization with 500 µl ice cold methanol (−20°C), for 5 min at 4°C. Subsequently, the samples were immunolabeled as described above.

Cells were mounted in 70% glycerol supplemented with DAPI. Imaging was performed using a LSM800 Zeiss confocal laser scanning microscope with a 63 × 1.4 NA oil immersion objective. Images were analyzed using FIJI‐ImageJ [39].

### 4.5 Live Cell Microscopy

10,000 moDCs cells were seeded in 100 µL RPMI1640 lacking phenol red and FBS in a four‐compartment glass‐bottom dish (627870; Greiner). 1–2 h after transfection, the cell medium was refreshed and cells were cultured overnight at 37°C and 5% CO_2_. The next day, 100,000 allogenic PBLs were added in each well. The samples were imaged at 37°C with a home‐built epi‐fluorescence microscope using a 490 nm laser for excitation, an Olympus 60× UAPO NA 1.49 Oil objective, and a Prime BSI Express Scientific CMOS camera (Photometrics). The microscope was controlled with µManager [40], and images were analyzed using FIJI‐ImageJ [39].

### 4.6 Silica Particles

Silica particles were from USA Silica (Min‐U‐Sil10) and incubated at ∼10 particles per cell for 1 h followed by fixation and immunofluorescence labelling.

### 4.7 Enzyme‐Linked Immunosorbent Assay (ELISA)

After overnight incubation, MLR culture supernatants were collected and the IFN‐γ cytokine level was assayed by Human IFN‐γ ELISA kit (Invitrogen,88‐7316) according to the manufacturer's instructions.

### 4.8 Flow Cytometry for Cell Surface Markers

Human moDCs were seeded overnight in 100 µL of RPMI medium with or without FBS and LPS at a density of 10^5^ cells/well in a 96‐well plates. The next day all cell samples were stained with Fixable Viability Dye eFluor780 (eBioscience, 1:2000 in cold PBS, 100 µL) on ice for 20 min followed by washing with PBS and staining for several surface expression markers MHC‐I, MHC‐II and CD86 by incubating 10^5^ cells in 30 µL/well with corresponding antibodies (Table 2) for 30 min at 4°C in the dark. Cells were washed three times with PBS prior to acquisition, and they finally were resuspended in 100 µL/well PBS and analyzed with flow cytometry (CytoFlex S flow cytometer, Beckman Coulter Inc., Indianapolis, IN, USA).

### 4.9 Flow Cytometry for Cell Death

Cryopreserved PBLs and moDCs of 4 donors were thawed, washed twice with PBS, pelleted at 300 xg for 5 min in 15 mL, and resuspended at 1 × 105 (PBLs) and 1 × 104 (moDCs) cells/50 µL in RPMI medium supplemented with 10% decomplemented human serum, 2 mM L‐glutamine, and 1% antibiotic‐antimycotic (RPMI‐HS‐L‐Glu‐AA medium). The allogeneic cocultures were made by mixing moDCs and PBLs from different donors in a 1:10 ratio in 100 µL in a 96‐well non‐adherent suspension culture plate. Mixtures of moDCs and PBLs from the same donor were used as negative control for T cell activation. All co‐cultures were incubated for 5 days at 37°C and 5% CO_2_. Before the fixation step, all cell samples were stained with Fixable Viability Dye eFluor780 (eBioscience, 1:2000 in cold PBS, 100 µL) on ice for 10 min followed by 100 µL/per well wash with PBS containing 1% BSA. The cells were stained for the CD3 receptor with 25 µL mouse anti‐CD3 APC‐conjugated antibody (eBioscience, Catalog # 17‐0038‐42, 1:100 in 1% BSA) on ice for 30 min. After washing with 100 µL PBS the cells were resuspended in 100 µL/well PBS and analyzed with flow cytometry. Flow cytometry was performed using a CytoFlex S flow cytometer (Beckman Coulter Inc., Indianapolis, IN, USA), and the data was analyzed using Novo Express software. The lymphocyte population was gated by using forward and side‐ward scatter (FSC; SSC), and the single cells were selected by plotting forward scatter area against forward scatter height (FSC‐A; FSC‐H). CD3‐negative cells were selected (APC^−^) and cell viability was assessed based on eFluor780 signal.

### 4.10 Transmission Electron Microscopy

Sapphire discs were incubated with polyethylamine (0.2% PEI) in H_2_O for 1 min. Discs were placed in a 24 ‐well dish, rinsed twice with H_2_O, and dried at room temperature. moDCs from donor 1 together with PBLs from donor 2were seeded in RPMI complete medium and allowed to interact for 2 h. Samples were immediately transferred to a LEICA EM ICE machine for high‐pressure freezing. Freeze substitution was performed in a Leica automated freeze substitution machine according to the following protocol: 33 h at −90°C with 4% uranyl acetate in methanol and osmium tetroxide the temperature was then raised to −20°C, held for 14 h, and then raised to 5°C. Embedding was started with acetone washes and infiltration of the samples with Epon 812 substitute like Glycid Ether, with hardener (MNA) and accelerator (DMP‐30) mixtures with increasing amounts of EPON. Samples were infiltrated with pure EPON overnight and then allowed to polymerize at 60°C for 2–3 days. Serial sections of 70 nm were cut with an automated tape collecting ultra‐microtome (Leica, UC6). Ultrathin sections were contrasted with uranyl acetate and lead citrate and examined under the Thermo Fisher Talos L120C Transmission Electron Microscope.

### 4.11 Statistical Analysis

For the statistical analysis in Figure 3D, data were tested for normality D'Agostino‐Pearson omnibus test and analyzed by paired 2‐sided student *t*‐test. Statistical significance was assumed for *p* < 0.05, and the p‐value and sample sizes are specified in the legend to the figure. Statistical analysis was performed in GraphPad Prism 10.

## Conflicts of Interest

The authors declare no conflicts of interest.

## Supporting information




**Supporting File**: adbi70077‐sup‐0001‐SuppMat.docx

**Supporting File**: adbi70077‐sup‐0002‐MovieS1.mp4

## Data Availability

We will deposit the data at the open repository Zenodo upon acceptance of the manuscript. Zenodo issues DOIs.
