# Supplementary material for: What Is the Role of Giant Endosomal Sorting Complexes Required for Transport (ESCRT) Structures in T Cell Activation?
Source: Adv Biol (Weinh). 2025 Nov 22;10(1):e00226. doi: 10.1002/adbi.202500226 (PMC12798694; doi:10.1002/adbi.202500226)
Supplement: Supplementary file 1 — Supporting File: adbi70077‐sup‐0001‐SuppMat.docx [file ADBI-10-e00226-s002.docx]

**Supplementary Information**

**What is the role of giant endosomal sorting complexes required for transport (ESCRT) structures in T cell activation?**

Anthi Psoma, Femmy C. Stempels, Rinse de Boer, Geert van den Bogaart

Department of Molecular Immunology, Groningen Biomolecular Sciences and Biotechnology Institute, University of Groningen, Groningen, the Netherlands

Contents:

-Supplementary Figure 1

-Supplementary Figure 2

-Supplementary Figure 3

-Supplementary Movie legend


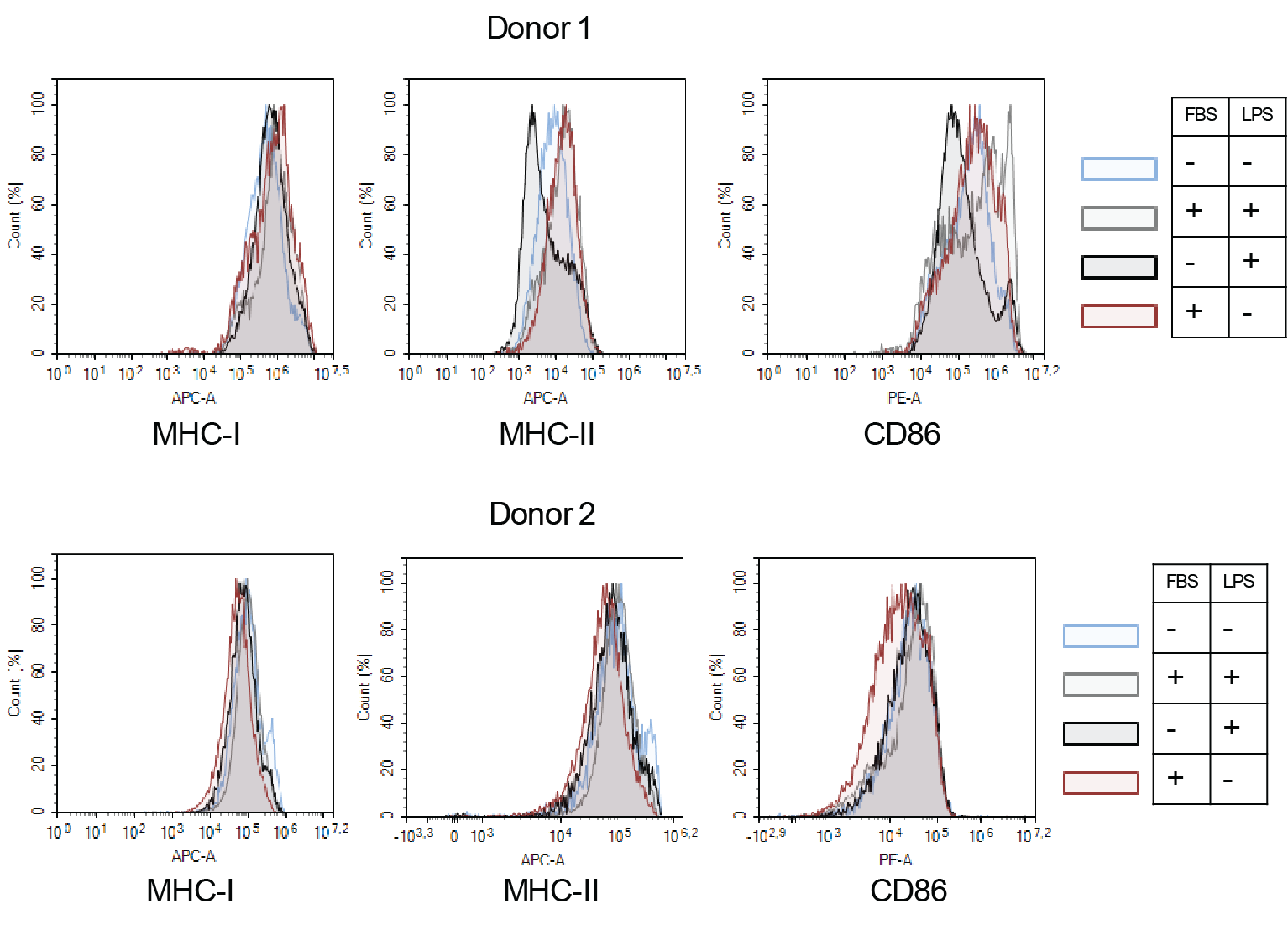


**Supplementary Figure 1. Presence of serum does not consistently affect surface levels of MHC class I, MHC class II and CD86.** MoDCs were cultured with RPMI medium with/ without FBS and with/without LPS. After 1 day of culture, expression levels of MHC-I, MHC-II,CD86 were analysed by flow cytometry. The graphs are for two different representative donors.


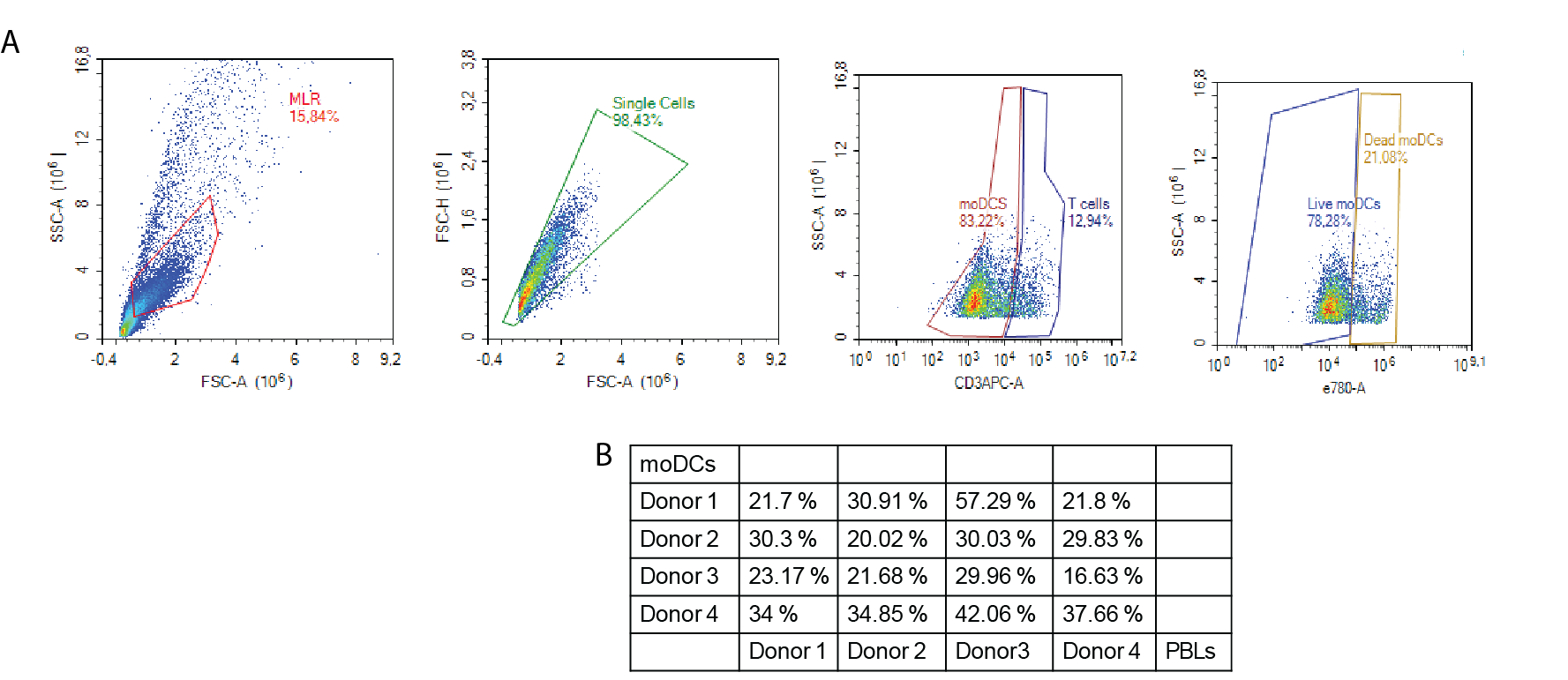


**Supplementary Figure 2. Mixed leukocyte reaction (MLR) results in cell death.** (**A**) Flow cytometry gating strategy used for the MLR with autologous (n=4 combinations) or allogenic pairs (n=12 combinations) of PBLs and moDCs. Cells were selected based on forward and side scatter, followed by collecting single cells. Next CD3-negative cells were selected, and cell death was quantified based on efluor780 signal. **(B)** Table presenting the percentage and the combinations of the donors for allogenic/autologous reactions.


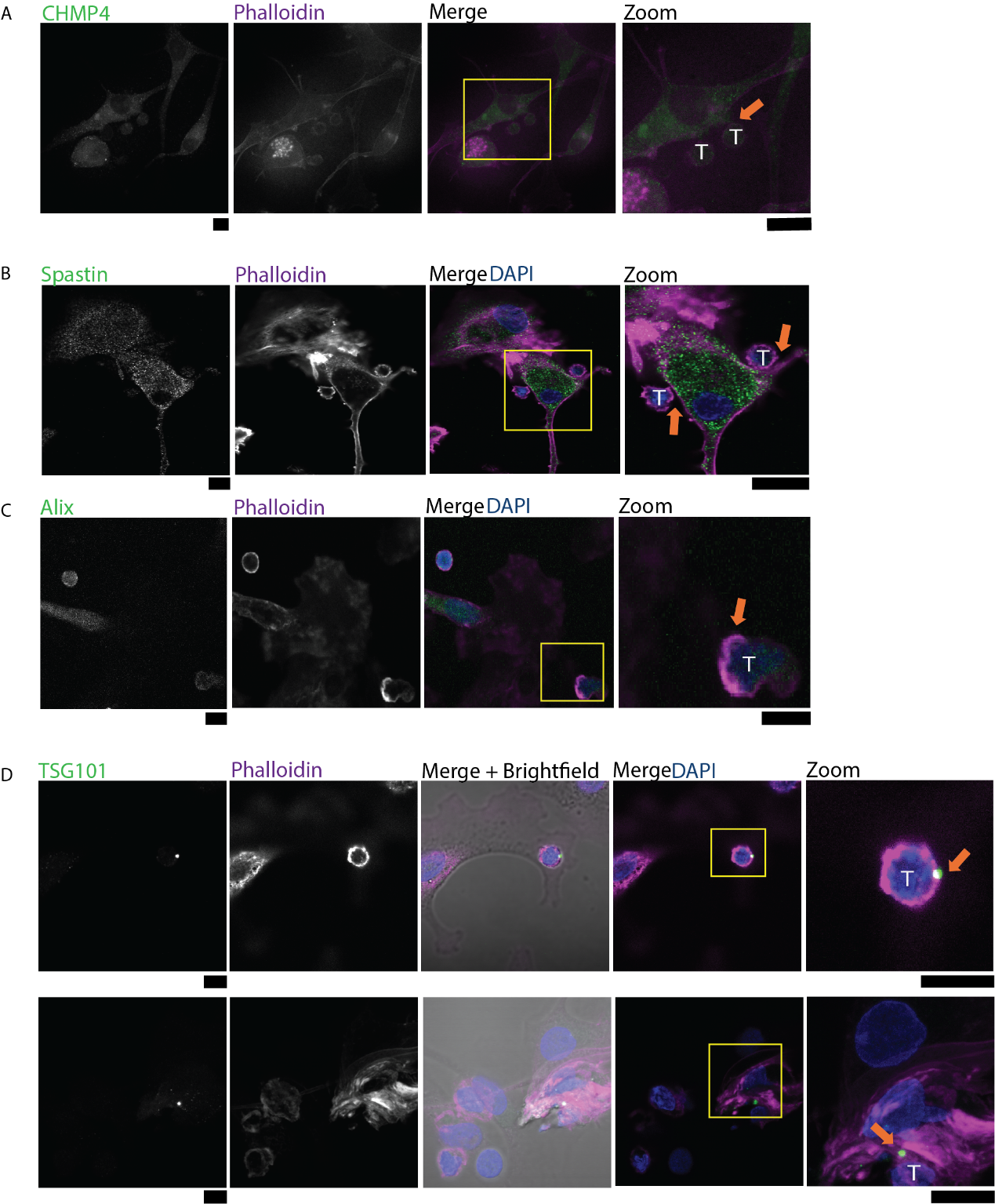


**Supplementary Figure 3 Giant ESCRT structures are not present at the IS with allogenic T cells. Only TSG101 occasionally accumulates at the IS between moDCs and PBLs. (A-C)** Representative confocal micrographs of moDCs cocultured together with allogenic PBLs (containing both CD4 and CD8 T cells) on glass supports without serum, and immunostained for CHMP4 (n=3 independent donors, 22 cells), Spastin (n=3 independent donors, 28 cells), or ALIX (n=3 independent donors, 16 cells). Green in merge: CHMP4, Spastin or ALIX; magenta: phalloidin; blue: DAPI. Yellow boxes: zoomed areas. **(D)** Confocal micrographs of moDCs cocultured with PBLs on glass supports without serum. Cells were immunostained for TSG101 (n=3 independent donors, 18 cells). Green in merge: TSG101; magenta: phalloidin; blue: DAPI. TSG101 accumulation at the IS was only occasionally observer for limited amount of cells of a single donor combination out of 3 tested. Orange arrows: position of the IS. 2 examples shown. Scale bars: 10 μm.

**Supplementary Movie 1 legend.** T**ime-lapse live cell imaging by fluorescence microscopy of** an moDC transfected with IST1-GFP (green) cultured together with allogenic T cells over a time period of 4 h and 43 min at 37^o^C, 63x objective. Scale bar 5 μm.
